# Successful application of chemosaturation with percutaneous hepatic perfusion in metastatic uveal melanoma patient progressing after systemic treatment options: a case report

**DOI:** 10.3389/fonc.2024.1355971

**Published:** 2024-04-10

**Authors:** Damla Gunenc, Ahmet Anil Ozluk, Utku Mahir Yıldırım, Paolo A. Ascierto, Burcak Karaca

**Affiliations:** ^1^ Department of Medical Oncology, Ege University Faculty of Medicine, Izmir, Türkiye; ^2^ Department of Interventional Radiology, Izmir University of Economics, Medicalpoint Hospital, Izmir, Türkiye; ^3^ Melanoma, Cancer Immunotherapy and Development Therapeutics Unit, Istituto Nazionale dei Tumori IRCCS “Fondazione G. Pascale”, Naples, Italy

**Keywords:** uveal melanoma, chemosaturation, immune checkpoint inhibitors, tebentafusp, liver, percutaneous hepatic perfusion

## Abstract

Uveal melanoma (UM) is a rare subtype of melanoma, accounting for less than 5% of all melanoma cases. Metastatic UM differs notably from cutaneous melanoma, exhibiting variations in etiology, prognosis, driver mutations, metastatic patterns, and poor responses to immune checkpoint inhibitors (ICI). Beyond local treatment options, such as resection, radiation therapy, and enucleation, and systemic treatments, such as ICIs, the approval of tebentafusp, a bispecific gp100 peptide-HLA-directed CD3 T-cell engager, marks a breakthrough in treating HLA-A*02:01 metastatic UM. Despite the advancements in treatment options, the long-term survival rates remain inadequate. We report a patient with metastatic UM who previously received ICI and progressed on tebentafusp treatment but subsequently exhibited a remarkable response to local treatment targeting liver metastasis. Such observations highlight the significance of exploring sequential therapeutic strategies for advanced UM, offering potential avenues to enhance treatment efficacy and patient prognosis.

## 1 Introduction

Uveal melanoma (UM) is a rare form of melanoma originating from melanocytes in the uvea (1). Caucasian population and 50-70 age group are at greater risk for UM, and occurrence before adulthood is uncommon (2). UM poses unique challenges due to its rarity and differences from cutaneous melanoma. Unlike cutaneous melanoma, UM is less associated with UV radiation exposure, characterized by a low mutation burden and a lack of ultraviolet (UV) mutational signatures, especially in posterior UMs (3).

Treatment involves various local therapies, including radiotherapy, phototherapy, and surgical resection, especially in early-stage cases. But, still, approximately half of UM patients progress to metastatic disease, frequently involving the liver due to hematogeneous metastatic behavior different from its cutaneous counterpart, which is usually associated with lymphatic spread.

ICIs revolutionized the prognosis for advanced cutaneous melanoma; however, they have demonstrated disappointing results in UM. Lower somatic mutation burden in UM, reduced immunogenicity, lower presence of neoantigens, and reduced programmed death-ligand 1 (PD-L1) expression, indicating immune evasion by tumor cells, have been proposed as potential reasons. Median progression-free survival (PFS) with ICIs ranges from 3-5.5 months, and median overall survival (OS) ranges from 12.7-19.1 months in phase 2 clinical trials (4, 5). Recently, tebentafusp, a bispecific peptide-HLA-directed T-cell engager, showed promising results with an estimated mOS of 21.7 months for HLA-A*02:01 positive patients with metastatic UM (6). However, the duration of response remains relatively short.

Recent advancements in local therapeutic approaches highlight the significance of hepatic-targeted treatments as a crucial area of research, especially in UM patients where liver metastases are commonly prevalent at the time of patient death, representing a significant challenge in treating metastatic disease. Various endovascular therapies offer promising strategies for addressing primary and metastatic hepatic malignancies, including bland arterial embolization, chemoembolization, radioembolization, and immunoembolization. Among these, chemosaturation with percutaneous hepatic perfusion (CS-PHP) has emerged as a minimally invasive, repeatable targeted hepatic therapy for UM metastases (7).

CS-PHP delivers melphalan directly to the hepatic artery to maximize the local concentration in the liver while minimizing systemic exposure and toxicities. Studies have reported encouraging response rates of up to 83% with improved local tumor control in hepatic metastases of uveal melanoma (8, 9). Furthermore, recent investigations demonstrated superior hepatic PFS rates in patients receiving CS-PHP compared to those treated with the best alternative care, encompassing arterial embolization, systemic chemotherapy, and supportive measures (10, 11).

However, there is a strong need for additional studies exploring therapeutic sequences in UM, especially for challenging cases progressing after limited systemic treatment options, where local treatment options might still offer potential for extended survival rates for these. Herein, we present our case report, aiming to share promising results of CS-HSP observed in a metastatic UM patient with predominantly liver metastases progressing after ICI and tebentafusp therapy.

## 2 Case report

A 40-year-old male patient was diagnosed with UM in February 2014, presenting with a history of blurry vision for approximately four months. Subsequently, a 12x9x10 mm lesion was detected in his right eye, and enucleation was performed. Optic nerve and sclera invasion were not observed. Whole body scanning showed no signs of distant metastasis.

The patient remained under surveillance without any signs of disease recurrence for seven years. However, in February 2021, two liver lesions measuring 42x30 mm and 12x10 mm were identified in liver segments VI-VII. He was decided to undergo metastasectomy for liver lesions, but unfortunately, surgery was terminated due to widespread millimetric metastases (**Supplementary Figure 1**). The liver biopsy confirmed UM metastasis. Hepatic angiography was performed to assess the suitability of transarterial radioembolization (TARE), but he was not found to be eligible for local treatment due to low uptake of Tc-99m-macroaggregated albumin (MAA).

He received weekly paclitaxel plus carboplatin chemotherapy. As conventional systemic chemotherapy is usually unsuccessful in metastatic UM, after six weeks of treatment, liver MRI revealed progression in the liver lesions measuring 45x31 mm, 17x7 mm, and a few millimetric metastatic lesions were observed again in the posterior segment level in the right lobe of the liver causing slight indentation in the capsule, leading to a referral to our clinic for further management.

Nivolumab treatment was initiated in May 2021, administering 240 mg doses every two weeks (Q2W) since tebentafusp was not available in our country at that time. After 6th cycle of the nivolumab, bi-phenotypic response was observed. While undergoing ICI treatment, we started discussions with a medical center in Italy for potential inclusion in the tebentafusp Early Access Program (EAP). After confirming HLA*02:01 positivity, the patient was eligible for the tebentafusp EAP.

In December 2021, MRI showed lesion progression in segments VI-VII previously reported 67x36 mm to 100x61mm, and millimetric metastases were found similar in size. The nivolumab treatment was discontinued after 18 cycles due to disease progression, and the patient was referred to Istituto Nazionale Tumori in Naples, Italy, for the tebentafusp EAP in February 2022. He received tebentafusp initially at 20 μg on day 1, 30 μg on day 8, 68 μg on day 15, and then 68 μg intravenously once weekly after that, as recommended in the protocol. He developed hair depigmentation, grade 2 rash, and cutaneous edema (**Supplementary Figure 2**) without any other severe adverse effects. Following three months of tebentafusp treatment, the most extensive liver lesion slightly decreased in diameter (96x59mm), and after six months of treatment, MRI showed approximately 30% necrosis in the most significant lesion. In the imaging conducted in December 2022, in addition to progression in the target lesion in the liver to 108x56mm, two new lesions were identified in segments 6 and 8. The patient continued weekly treatment for 50 cycles, and therapy was discontinued due to confirmed progressive disease detected by subsequent imaging 8 weeks later that showed progression of his liver lesions and newly developed 26 mm lesion adjacent to the kidney, along with suspicious millimetric bone metastases in the lumbar and sacral vertebral bodies.

The patient was referred to our medical department for best supportive care. At the first administration to our clinic, the patient was in an excellent performance status (ECOG 0) and greatly desired treatment. He was not suitable for some local treatment options such as surgery and TARE. CS-PHP, being a relatively uncommon practice in our country and performed only in a few specialized centers, had not been utilized in the patient’s previous treatment lines. Considering that the liver lesions will determine the survival of this patient, we decided to treat the patient using melphalan-delivered CS-PHP, specifically targeting the liver lesions.

**Figure 1:**
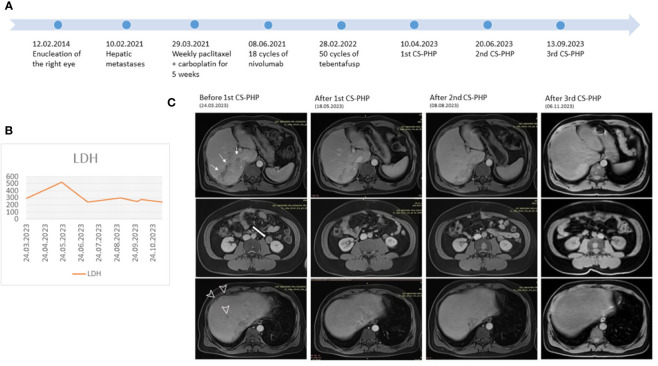
**(A)** Course of the disease **(B)** LDH levels during CS-PHP procedures **(C)** Serial MRI images before and after each CS-PHP procedure showing the biggest lesion in the liver (thin arrows), lesion adjacent to kidney (thick arrow) and scattered liver metastases (arrowhead).

Abdomen MRI before the first CS-PHP showed a 115x66 mm solid metastatic lesion without necrosis, involving liver segments 6-7 and growing towards the inferior vena cava, as well as many scattered metastatic lesions in both lobes of the liver, the largest of which measured 16 mm in diameter and a 26 mm stable soft tissue metastasis adjacent to the left kidney. After his first procedure in April 2023, the most extensive lesion, measuring 115x66 mm, decreased to 83x41 mm, most of the millimetric lesions disappeared; interestingly, the 26 mm lesion adjacent to the left kidney reduced to 18 mm. In June 2023, the second CS-PHP procedure was performed. Further decrease in the size of the most extensive lesion was observed from 83x41 mm to 70x28 mm, with the majority exhibiting necrosis, and 16 mm lesion in segment 8 decreased to 12 mm. After the third application of CS-PHP without any severe adverse effect, the most extensive liver lesion measured stable as 67x34 mm and only two remaining lesions were observed as 6 mm and 3 mm in segment 8 (**Figure 1**). Diffusion-weighted MR images revealed complete necrosis of liver lesions, and there was no newly developed liver lesion. Control PET/CT showed almost complete metabolic response in liver lesions, multiple stable metastatic bone lesions with the acetabular lesion showing partial metabolic response, and a few newly developed lesions in the humerus and columna vertebralis (**Supplementary Figure 3**). He has initiated treatment with zoledronic acid for new bone lesions.

The MRI conducted in January 2024 revealed findings suggestive of progression characterized by an increase in the area with high T1 signal intensity, which was previously almost necrotic. Additionally, the lesion previously measured at 12 mm has progressed to 32 mm, and on the left lobe side, new lesions measuring 6 mm, 5 mm, and 4 mm have developed. Our patient showing progression after multiple-line systemic treatments achieved a hepatic PFS of 9 months after the first CS-PHP. After 35 months since the initial detection of metastasis, the patient remains asymptomatic with an ECOG 0 performance status. Due to limited treatment options, a fourth CS-PHP has been scheduled for the newly developed liver lesions.

## 3 Discussion

Treatment options for metastatic UM include tebentafusp, ICIs, and liver-directed locoregional therapies. Therapies targeting BRAF or KIT are not indicated in UM in the absence of the corresponding mutations. MEK inhibition with trametinib and selumetinib has been tested and shows minimal clinical effect (12, 13). ICIs can still be an option in cases where local ablative options are not feasible, and the patient is HLA A*02:01 negative. Tebentafusp is the first systemic treatment for HLA A*02:01 positive UM ever to show a significant survival benefit in a prospective randomized trial with OS at one year of 73% compared to 59% in the control arm of investigator’s choice of therapy with single-agent pembrolizumab, ipilimumab or dacarbazine (6).

Due to the strong hepatotropism of its metastatic pattern, UM patients might usually benefit from liver-directed treatments, and these local interventions can be a valid alternative to systemic therapy, such as hepatic intra-arterial chemotherapy, hepatic embolization, radiofrequency ablation, or stereotactic radiation therapy. Among these liver-directed treatment options, limited clinical efficacy, significant systemic toxicity, and safety concerns have suspended the widespread use of isolated hepatic perfusion for administering high-dose chemotherapy. But, a recent advancement overcame these challenges using isolated hepatic perfusion in a method known as “chemosaturation”, utilizing melphalan to deliver highly concentrated chemotherapy directly to the liver. This method provides high concentrated chemotherapy to the hepatic artery through a catheter inserted in the femoral artery. A double-balloon catheter is placed at the atrium–IVC junction and infrahepatic IVC above the renal veins to isolate hepatic venous circulation. Hepatic venous outflow is diverted from the lumen of the double-balloon catheter to extracorporeal circulation. Blood returning from the hepatic veins containing melphalan is passed through special filters that catch 95-96% of melphalan and remove it from hepatic venous blood (14).

In the first phase III trial, 83 UM and 10 cutaneous melanoma patients were randomly assigned to receive either CS-PHP (n = 44) or best alternative care (BAC) (n = 49). In this trial, first-generation filters were used, which are less efficient in removing melphalan than the current second-generation filters. BAC consisted of investigators’ choice of treatment, most frequently using temozolamide, and 18.4% of patients received only best supportive care. Hepatic progression-free survival (hPFS) was the primary endpoint of the trial, demonstrating 7 months in CS-PHP arm compared to 1.6 months in BAC arm (p < 0.0001). mPFS was 5.4 months for CS-PHP and 1.6 months for BAC (p < 0.0001). Median OS did not significantly differ between CS-PHP (10.6 months) and BAC (10.0 months), possibly due to the crossover of 57.1% of BAC patients to CS-PHP after progression. The hepatic overall response was 36.4% for CS-PHP and 2% for BAC (10).

Recently, a multicenter phase III FOCUS study for hepatic dominant UM presented updated results at ASCO 2022 (15). The study was initially started as a randomized trial comparing CS-PHP to BAC. However, it was converted to a single-arm CS-PHP study due to enrollment concerns in the BAC arm. 144 patients were enrolled with 91 PHP and 32 BAC patients receiving treatment. The ORR in the CS-PHP population was 35.2% and 12.5% in the BAC population. While the duration of response (DoR), PFS, and OS data still remain immature, the reported median DoR on CS-PHP was 14 months and not calculable for BAC patients. The median PFS was 9.03 vs. 3.06 months, and OS was 20.53 vs. 14.06 months for CS-PHP and BAC populations, respectively.

In addition to the above-mentioned prospective trials, several retrospective cohorts and case series supported the clinical feasibility and effectiveness of this method. While this innovative approach has demonstrated promising results in patients with metastatic UM (**Table 1**), further exploration through well-balanced multi-center prospective trials is still needed to enhance clearer generalizability.

**Table 1:** CS-PHP cohort studies for metastatic uveal melanoma.

Study	Study Type	Sample Size (n)	ORR (%)	Grade 3-4 Toxicity
Hughes et al. (10)	Phase III	93 (PHP, n = 44 vs. BAC, n = 49) (UM, n = 83; cutaneous melanoma, n = 10)	36.4%; all of PR	85.7% Neutropenia 80% Thrombocytopenia 62.9% Anemia 17.1% Febrile neutropenia 14.3% Bilirubin elevation
Karydis et al. (16)	Retrospective	51	49%; 43.1% PR 5.9% CR	37.5% Non-hematologic 31.3% Neutropenia 31.3% Thrombocytopenia 29.4% Anemia
Artzner et al. (11)	Retrospective	16	60%; all of PR	14% Leukopenia 14% Thrombocytopenia
Brüning et al. (17)	Retrospective	19	53% PR 47% SD	Not specified
Modi et al. (18)	Retrospective	81	60.5%; 51.9% PR 8.6% CR	13.3% Anemia 13.3% Neutropenia 12% Thrombocytopenia 7.2% Coagulopathy
Dewald et al. (19)	Retrospective	66	59%	24.8% Thrombocytopenia 7.6% Hepatic toxicity 4.1% Leukopenia
Meijer et al. (20)	Phase II	35	72%; 69% PR 3% CR	84.8% Lymphocytopenia 75.6% Leukopenia
Tong et al. (21)	Retrospective	101	59.5%; 54.5% PR 5% CR	24% Leukopenia 28.4% Thrombocytopenia 14.2% Hepatic toxicity 7.6% Anemia
Zager et al. (15)	Phase III (FOCUS)	144 (switched to single-arm PHP, n = 91)	35.2%	42.6% Serious TEAE 14.9% Thrombocytopenia 10.9% Neutropenia 4.2% Leukopenia

BAC, best alternative care; CR, complete response; PHP, percutaneous hepatic perfusion; PR, partial response; ORR, Overall response rate; OS, overall survival; TEAE, Treatment emergent adverse events; UM, uveal melanoma.

When patient selection and treatment goals are appropriately determined, CS-PHP may offer distinct advantages over other local treatment options. The ability of CS-PHP to affect the entire liver makes it a viable option for larger and also non-visible lesions. Additionally, embolization of hepatic artery branches during transarterial chemoembolization (TACE) can lead to ischemic injury, while selective internal radiation therapy (SIRT) poses a risk of radiation injury to healthy liver tissue. Furthermore, CS-PHP may be more suitable for patients with recurrent or refractory disease, allowing for repeat procedures.

In a retrospective study, including 29 patients who underwent TACE using cisplatin, the ORR was 21% and median PFS was 6 months. Adverse events of grade ≥3 included aspartate aminotransferase (AST) elevation in 34.5%, alanine aminotransferase (ALT) elevation in 51.7%, and serum creatinine elevation in 3.4% (22). In the largest prospective study of TACE, 24 patients were evaluated. The ORR was 20%, with a median OS of 5 months. Eight patients experienced grade ≥3 complications (23). In a retrospective study evaluating the efficacy and safety of SIRT in patients with systemic therapy-resistant metastatic uveal melanoma, the ORR remained limited at 17.9%. 2 (7%) of 28 patients receiving SIRT suffered mortality due to hepatic failure within 1 month (24). A single-center study, comparing SIRT and CS-PHP for hepatic metastasized uveal melanoma included 62 patients (SIRT, n=34 vs. CS-PHP, n=28). The disease control rate was 18% for SIRT and 30% for CS-PHP. CS-PHP showed a significant OS benefit as compared with SIRT (median 516 days vs. 300.5 days, p = 0.006) (25).

To our knowledge, tebentafusp treatment leads to a high proportion of T-cell infiltration and increased cytokine release in the tumor microenvironment (TME) (26, 27). Some adverse effects related to tebentafusp treatment, such as skin rash or pruritus, are probably a result of the interaction between T cells and gp100-expressing melanocytes (28). Patients who experienced rash within the first week of tebentafusp treatment had significantly better survival rates. Also, several studies showed that inflammatory TME is associated with chemosensitivity (29, 30).

On the other hand, the immunomodulatory effects of chemotherapeutics are currently being investigated, and their clinical use combined with chemotherapy is becoming widespread based on this hypothesis (31). Considering the potentializing effect of either combined or sequential use of these treatments on each other’s effectiveness, we emphasize that the response of our patient, who had previously received both ICI and tebentafusp, should be evaluated within this framework. The treatment response in the lesion adjacent to the kidney and some bone lesions that do not receive local treatment may become more explainable in the light of this information.

## 4 Conclusion

One of the significant issues in UM is high risk for distant recurrence after local treatment, with up to 50% of patients developing distant metastases. In 90% of cases, the liver is the first site of metastasis (32). Our case underlines that CS-PHP is an effective salvage treatment for liver-dominant metastatic UM even after progression after ICI and tebentafusp treatment. Due to being a rare melanoma and limited treatment options, there is a considerable unmet clinical need of shared experience for combining and sequencing treatments for metastatic UM patients.

## Data availability statement

The original contributions presented in the study are included in the article/**Supplementary Material**. Further inquiries can be directed to the corresponding author.

## Ethics statement

Written informed consent was obtained from the patient for the publication of any potentially identifiable images or data included in this article. The study was performed in accordance with the Declaration of Helsinki, Good Clinical Practice and applicable regulatory requirements.

## Author contributions

DG: Conceptualization, Writing – original draft, Writing – review & editing. AO: Writing – original draft, Writing – review & editing. UY: Supervision, Writing – original draft, Writing – review & editing. PA: Writing – original draft, Writing – review & editing. BK: Conceptualization, Supervision, Writing – original draft, Writing – review & editing.

## Supplementary material

Supplementary Figure 1 Widespread millimetric liver metastasis at the time of the diagnosis of metastatic disease.

Supplementary Figure 2 Rash and facial edema during tebentafusp treatment.

Supplementary Figure 3 Diffusion-weighted MRI images before **(A)** and after **(B)** 3rd CS-PHP procedure. **(C)** PET/CT fusion image showing no FDG uptake in the most extensive liver lesion after the 3rd CS-PHP procedure.
