# Supplementary figures and images for: Successful application of chemosaturation with percutaneous hepatic perfusion in metastatic uveal melanoma patient progressing after systemic treatment options: a case report
Source: Front Oncol. 2024 Apr 10;14:1355971. doi: 10.3389/fonc.2024.1355971 (PMC11040682; doi:10.3389/fonc.2024.1355971)

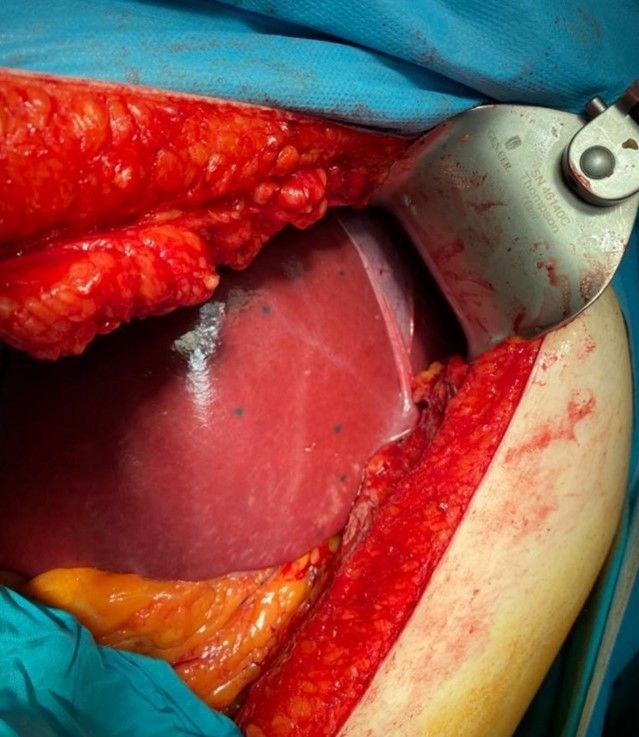

Supplement: Supplementary Figure 1 — Widespread millimetric liver metastasis at the time of the diagnosis of metastatic disease. [file Image_1.jpg]

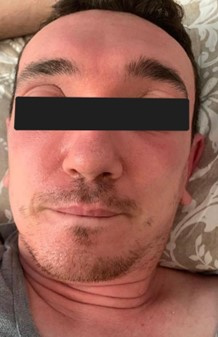

Supplement: Supplementary Figure 2 — Rash and facial edema during tebentafusp treatment. [file Image_2.jpg]

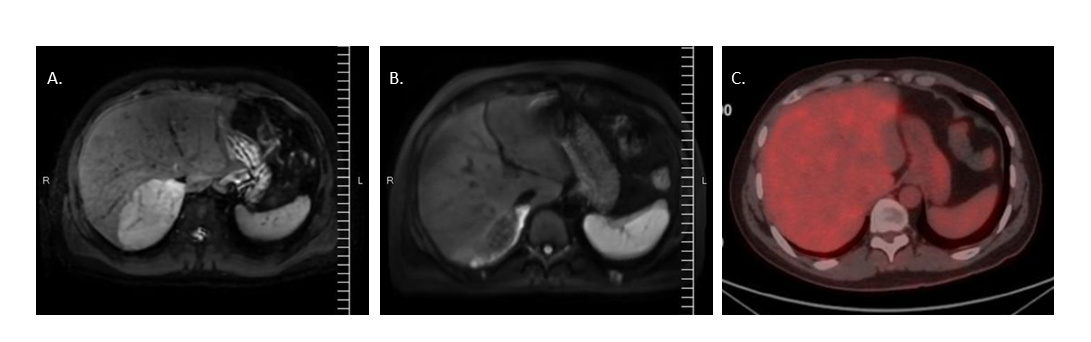

Supplement: Supplementary Figure 3 — Diffusion-weighted MRI images before (A) and after (B) 3rd CS-PHP procedure. (C) PET/CT fusion image showing no FDG uptake in the most extensive liver lesion after the 3rd CS-PHP procedure. [file Image_3.jpg]
